# Gene Expression-Based Functional Differences between the Bladder Body and Trigonal Urothelium in Adolescent Female Patients with Micturition Dysfunction

**DOI:** 10.3390/biomedicines10061435

**Published:** 2022-06-17

**Authors:** Natalia Zeber-Lubecka, Maria Kulecka, Katarzyna Załęska-Oracka, Michalina Dąbrowska, Aneta Bałabas, Ewa E. Hennig, Magdalena Szymanek-Szwed, Michał Mikula, Beata Jurkiewicz, Jerzy Ostrowski

**Affiliations:** 1 Department of Gastroenterology, Hepatology and Clinical Oncology, Centre of Postgraduate Medical Education, Roentgena 5, 02-781 Warsaw, Poland; nzeber@cmkp.edu.pl (N.Z.-L.); mkulecka@cmkp.edu.pl (M.K.); ehennig@cmkp.edu.pl (E.E.H.); 2 Department of Genetics, Maria Sklodowska-Curie National Institute of Oncology, Roentgena 5, 02-781 Warsaw, Poland; michalina.dabrowska@pib-nio.pl (M.D.); aneta.balabas@pib-nio.pl (A.B.); michal.mikula@pib-nio.pl (M.M.); 3 Department of Pediatric Surgery, Children’s Hospital, Centre of Postgraduate Medical Education, Marii Konopnickiej 65, 05-092 Dziekanow Lesny, Poland; katarzyna.zaleska@szpitaldziekanow.pl (K.Z.-O.); magdalena.szymanek-szwed@cmkp.edu.pl (M.S.-S.)

**Keywords:** bladder body urothelium, micturition dysfunction, microbiome, trigonal urothelium

## Abstract

The aim of this study is to determine the molecular differences between the urothelial transcriptomes of the bladder body and trigone. The transcriptomes of the bladder body and trigonal epithelia were analyzed by massive sequencing of total epithelial RNA. The profiles of urothelial and urinal microbiomes were assessed by amplicon sequencing of bacterial 16S rRNA genes in 17 adolescent females with pain and micturition dysfunction and control female subjects. The RNA sequencing identified 10,261 differentially expressed genes (DEGs) in the urothelia of the bladder body and trigone, with the top 1000 DEGs at these locations annotated to 36 and 77 of the Reactome-related pathways in the bladder body and trigone, respectively. These pathways represented 11 categories enriched in the bladder body urothelium, including *extracellular matrix organization*, *the neuronal system*, and 15 categories enriched in the trigonal epithelium, including *RHO GTPase effectors*, *cornified envelope formation*, and *neutrophil degranulation*. Five bacterial taxa in urine differed significantly in patients and healthy adolescent controls. The evaluation of their transcriptomes indicated that the bladder body and trigonal urothelia were functionally different tissues. The molecular differences between the body and trigonal urothelia responsible for clinical symptoms in adolescents with bladder pain syndrome/interstitial cystitis remain unclear.

## 1. Introduction

The urinary bladder develops from different embryological tissues, resulting from epithelial-mesenchymal interactions that are responsible for normal smooth muscle development and directing urothelial differentiation [1,2]. The bladder can be divided into the body and the base, with the latter consisting of the trigone, a zone located between the openings of the ureters and urethra, and the bladder neck. The trigone laminar architecture contains more connective tissue than the body, and its musculature exhibits more spontaneous myocyte activity [3]. The trigone is innervated [4,5,6,7] sympathetically and parasympathetically, with a synergy between adrenergic and muscarinic systems, thus playing roles in micturition and pain perception [8,9,10]. In contrast, the body urothelium deploys protective membrane plaques comprised of uroplakin proteins [10,11], accompanied by the expression of sensory proteins that regulate detrusor contractility [8]. These functional differences may result from incomplete differentiation of the trigonal epithelium [12,13,14]. Epithelial regeneration in response to injury or infection has been found to depend on the presence of stem cells and progenitor cells in the basal and intermediate layers, with higher numbers of progenitor cells detected in the trigonal urothelium [15,16,17].

The urothelium is a transitional epithelium consisting of three to seven cell layers: a basal layer, one or more intermediate layer(s), and a superficial or apical layer made up of large hexagonal cells called “umbrella cells”. Although normal trigonal epithelium does not differ morphologically from normal bladder body epithelium, they are differently involved in bladder filling, micturition, and the maintenance of bladder homeostasis [18]. Changes in urothelial functions, such as dysfunctional micturition and pain perception, may accompany different bladder disorders, including cancer, urinary infections, and bladder pain syndrome/interstitial cystitis (BPS/IC) [17,19,20].

Few studies to date have evaluated the molecular basis of morphological and functional changes in the urothelium of the bladder body and trigone, with most of these studies analyzing changes in the expression of single genes. Genes related to immune and inflammatory responses were found to be overexpressed in bladder tissues of patients with BPS/IC [2,21,22,23]. Moreover, the genes involved in maintaining the integrity of the bladder epithelial barrier were found to be altered in patients with BPS/IC. For example, the expression of uroplakin 3 was lower, whereas that of TNF-inducible gene 6 (*TSG-6*) was higher in the bladders of subjects with BPS/IC than in control subjects [8]. In addition, the expression of the *WNT* gene was found to be higher in patients with Hunner-type IC than in those with non-Hunner-type IC [3]. Disruption of the glycosaminoglycan (GAG) layer covering the urothelium may lead to the development of “a syndrome of leaking epithelium,” facilitating the penetration of irritants into bladder tissue, thereby causing chronic inflammation, and indirectly affecting the interplay between muscarinic and nicotinic receptors in the urothelium [9,10].

A cystoscopy of 254 children, 248 girls and 6 boys, who underwent diagnostic evaluation at our Department of Pediatric Surgery and Pediatric Urology [24] for abnormal bladder function and recurrent urinary tract infections in early childhood between 2005 and 2018 showed that most had fluffy surfaces in the bladder triangle and neck, accompanied by trigonal non-keratinizing squamous metaplasia. Urodynamic examination showed abnormal functioning of the urinary bladder, particularly hyperactivity of the detrusor muscle and reduced bladder capacity. Immunological tests showed reductions in the immunoregulatory functions of T lymphocytes, increases in the immunogenic activity of monocytes, and increased pro-inflammatory immunogenic activity of the immune system. Moreover, girls had low concentrations of progesterone in the luteal phase. Despite these findings, the understanding of micturition dysfunctions in these pediatric patients remains limited.

To better understand the molecular differences between the urothelium of the bladder body and trigone in adolescent girls with pain and micturition dysfunction, their transcriptomes were analyzed by massive sequencing of the total epithelial RNA. In addition, the profiles of urothelial and urinal microbiomes were assessed by amplicon sequencing of bacterial 16S rRNA genes. Although molecular analyses indicated that the bladder body and trigonal urothelia were functionally different tissues, sequencing-based analysis showed no differences in their urothelial microbiomes and indicated that chronic bacterial infections were not the main cause of these molecular changes.

## 2. Materials and Methods

### 2.1. Ethics Statement

All enrolled patients and control subjects were Caucasians. The study was approved by the local ethics committee (Centre of Postgraduate Medical Education, Warsaw, Poland, project ID: 28/PB/2020), and all participants and their parents provided written informed consent. The study protocol conforms to the ethical guidelines of the 1975 Declaration of Helsinki. Written informed consent for participation in this study was obtained from each participant before the examination.

### 2.2. Subjects and Samples Collection

Seventeen adolescent female patients, aged 12 to 18 years (median age, 16 years), admitted to the Department of Pediatric Surgery and Pediatric Urology for cystoscopy were recruited. All 17 suffered from a range of urological symptoms, including erythrocyturia, pollakiuria, dysuria, difficulty in initiating micturition, urinary retention, urinary incontinence, and nocturia, accompanied by nonspecific abdominal pain and pain during micturition, often requiring analgesic treatment. All but one patient underwent a urodynamic study (UDS); of these, in two cases the urodynamic examination was normal, while in the remaining patients, detrusor overactivity with increased sensation in the bladder was found. The ultrasound examination (USG), magnetic resonance imaging (MRI), computed tomography (CT), cystourethrography (CUM), and dynamic renoscintigraphy of kidneys (RSC) were also performed during the diagnostic procedures. In five patients, vesicoureteral reflux was diagnosed; of these, three and two patients underwent endoscopic and surgical treatment, respectively. In two patients, urolithiasis was also diagnosed, and in two others, hydronephrosis was diagnosed. All had previously experienced recurrent infections of the urinary tract, but their urine cultures were negative at admission. Time from the onset of symptoms to the current visit ranged from 1 to 7 years (median: 2 years). All underwent review cystoscopy under general anesthesia using a cysto-resectoscope (compact-type rigid cystoscope 8/9 F; 14 I 17 F, equipped with a telescope and cold cup biopsy forceps). Macroscopic examination in all subjects showed evidence of hyperemia, edema, and thickened epithelium of varying degrees of severity, ranging from weak to moderate, at the trigonal area of the urinary bladder. All the patients received Nitrofurantoin on the day preceding the cystoscopy and for 7 days after the procedure.

Two small biopsy specimens were obtained from the posterior wall of the urinary bladder of each subject: one for analysis of gene expression and the other for microbiome assessment. In addition, three biopsy specimens were obtained from the trigonal area, showing epithelial macroscopic abnormalities: one for histopathological examination, one for transcriptomic analysis, and one for metagenomic assessment.

The biopsy specimens used for histopathological examination were fixed in 10% formaldehyde, serially sectioned, and stained with hematoxylin and eosin. These samples were subsequently examined by an experienced pathologist, with the results shown in Table 1.

**Table 1:** Results of urodynamic studies and histopathological findings in biopsy specimens of the trigonal urothelium taken from 17 adolescent girls with urological symptoms.

Sample ID	Age (Years)	Results of Urodynamic Studies	Results of Histopathological Examination
1B	12	Increased sensation in the bladder. High maximal pressure in the bladder during micturition.	Reactive changes in the urothelial epithelium and underlying florid proliferation of von Brunn’s nests
2B	17	Not tested	Segmental non-keratinized squamous metaplasia without inflammatory infiltrates
3B	14	Mild detrusor overactivity with increased sensation in the bladder	Slight chronic inactive inflammation of the urothelial epithelium with reactive changes and focal squamous non-keratinized metaplasia
4B	13	Normal	Moderate chronic active inflammation and swelling of the urothelial epithelium with reactive changes
5B	17	Increased sensation in the bladder with reduced bladder capacity. Lower urinary tract dysfunction with urgency sensors.	Focal chronic active inflammation of the urothelial epithelium with reactive changes of the urothelial epithelium and focal non-keratinized squamous metaplasia
6B	14	Increased sensation in the bladder with reduced bladder capacity.	Moderate chronic active inflammation of the urothelial epithelium and non-keratinized squamous metaplasia
7B	17	Increased sensation in the bladder with reduced bladder capacity.	Chronic inactive inflammation of the urothelial epithelium and visible non-keratinized squamous metaplasia
8B	18	Detrusor overactivity with increased sensation in the bladder. Reduced bladder capacity.	Non-keratinized squamous metaplasia *
9B	17	Urethral flow with bladder obstruction features.	Non-keratinized squamous metaplasia
10B	13	Urethral flow with bladder obstruction features.	Reactive changes of the urothelial epithelium and underlying florid proliferation of von Brunn’s nests
11B	16	Detrusor overactivity with increased sensation in the bladder. Reduced bladder capacity.	Normotypic urothelial epithelium with swelling of the stroma and sparse infiltrates of lymphoid cells
12B	14	Detrusor overactivity with increased sensation in the bladder. Decreased bladder capacity.	Normotypic urothelial epithelium with swollen and bloodshot stroma
13B	16	Detrusor overactivity with increased sensation in the bladder. Reduced bladder capacity.	Non-keratinizing squamous metaplasia
14B	17	Detrusor overactivity with increased sensation in the bladder. Reduced bladder capacity.	Non-keratinized squamous metaplasia with poor lymphocyte infiltrates
15B	13	Detrusor overactivity with increased sensation in the bladder. Reduced bladder capacity.	Non-keratinized squamous metaplasia and cystitis glandularis
16B	14	Detrusor overactivity with increased sensation in the bladder.	Focal active inflammation of the urothelial epithelium and focal non-keratinized squamous metaplasia
17B	17	Detrusor overactivity with increased sensation in the bladder	Focal chronic inactive inflammation of the urothelial epithelium and squamous non-keratinized metaplasia

**Figure 1:**
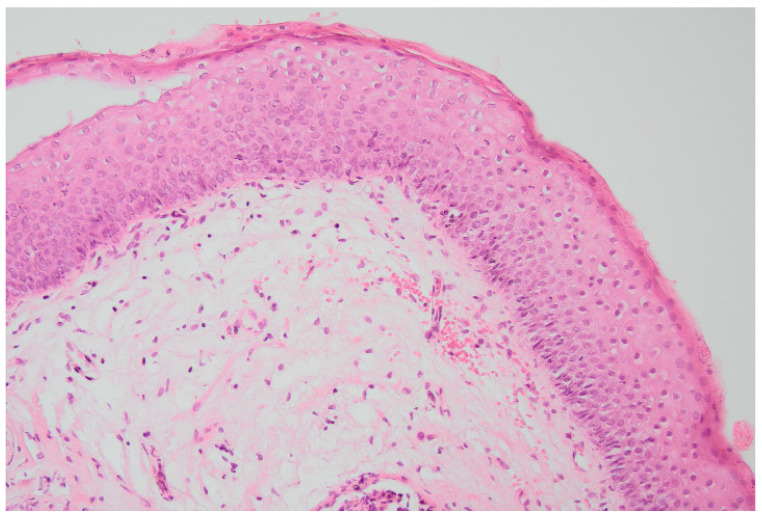
Histopathology examination showing non-keratinizing stratified squamous epithelium.

* An example of non-keratinized squamous metaplasia found at the triangle of the bladder (Figure 1).

Morning urine samples (sampled from the middle stream) were collected in sterile containers from the 17 studied patients (the day before the cystoscopy was performed), from female members of their families (13 mothers and 2 sisters) living in the same household, and from 20 adolescent female controls aged 11 to 17 years (median age, 14 years). None of the patients, or control subjects, presented clinical symptoms of urinary tract infection at the time of urine sampling, or received antibiotics during the 72 days before urine collection.

The biopsy specimens and urine samples used for total RNA and 16S rRNA sequencing were deep-frozen immediately, kept at −80 °C, and then transported to the Department of Genetics on dry ice.

### 2.3. RNA Isolation, Sequencing, and Data Analysis

Total RNA was isolated from urothelial specimens with mirVana™ PARIS™ RNA and Native Protein Purification Kit (Thermo Fisher Scientific, Waltham, MA, USA), according to the manufacturer’s instructions. The concentration and purity of the isolated RNA samples were assessed using a NanoDrop™ 2000 Spectrophotometer (Thermo Fisher Scientific, Waltham, MA, USA), while the integrity of these samples was assessed with an Agilent RNA 6000 Nano Kit on a 2100 Bioanalyzer (Agilent Technologies, Santa Clara, CA, USA).

RNA-Seq libraries were prepared and sequenced as described [25]. Signal processing and base calling were performed using Torrent Suite version 5.10. Reads were mapped to the hg19 AmpliSeq Transcriptome version 1 genome. Read counts per gene were obtained with HTSeq-count version 0.615 using default parameters. Gene expression was normalized, and differential gene expression was estimated using DESeq2 version 1.30, with default parameters and options. Genes with adjusted *p*-value (*p*adj-value) ≤ 0.05 were considered differentially expressed. Overrepresentation of Reactome terms (REACTOME_Pathways_20.11.2017 base) was determined using the Cytoscape platform version 3.6.1 with the ClueGo application. Heatmaps were visualized using the R package ComplexHeatmap version 2.7.6.1009. The RNA-Seq data have been deposited as BAM files in the European Nucleotide Archive (Accession number PRJEB50164).

### 2.4. DNA Extraction, 16S rRNA Sequencing, and Data Analysis

Genomic DNA was extracted and purified from biopsy specimens of bladder urothelium using QIAamp DNA Mini Kits (QIAGEN, Hilden, Germany) and from urine samples using PureLink™ Microbiome DNA Purification Kits (Thermo Fisher Scientific, Waltham, MA, USA). DNA concentrations were measured on a NanoDrop™ 2000 Spectrophotometer (Thermo Fisher Scientific, Waltham, MA, USA). 16S rRNA gene libraries were sequenced on an Ion Torrent Personal Genome Machine (PGM) platform (Thermo Fisher Scientific, Waltham, MA, USA) using Ion PGM™ Hi-Q™ View and Hi-Q™ View OT2 Sequencing Kits. Bacterial 16S rRNA libraries were prepared using an Ion 16S™ Metagenomics Kit, which allows a consensus view across six regions (V2, V3, V4, V6–7, V8, and V9), and an Ion Plus Fragment Library Kit, as previously described [26].

Unmapped BAM files were converted to FASTQ using Picard’s SamToFastq [27]. Additional steps of the analysis were performed using Mothur version 1.43 software [28]. FASTQ files were converted to the FASTA format. For analyses, only sequences 200–300 bp in length, with an average base quality of 20 in a sliding window of 50 bases and a maximum homopolymer length of 10, were kept. Chimeric sequences were identified with the VSEARCH chimera detection algorithm using default parameters [29], with internal sequence collection as the reference database. Chimeric sequences were removed, and the remaining 16S rRNA sequences were classified using the Wang method and the SILVA bacterial 16S rRNA database [30] for reference (release 138), at an 80% bootstrap cut-off. Data were normalized with metagenomeSeq [31], and differential taxa abundance was assessed with a mixed effects model for paired samples and a metagenomeSeq zero-inflated log-normal model for comparison with controls. The presence or absence of differences in taxa were determined with Fisher’s exact test. The non-parametric Shannon diversity index and the Chao1 richness index were determined with Mothur, with differences in values of indices assessed using the Mann–Whitney U-test or Wilcoxon’s paired test, as appropriate. Bray–Curtis indices and principal coordinate analysis (PCoA) analyses were performed with MEGAN5 [32]. FDR-adjusted [33] *p*-values ≤ 0.05 were considered statistically significant.

## 3. Results

### 3.1. Transcriptomic Profiling

**Figure 2:**
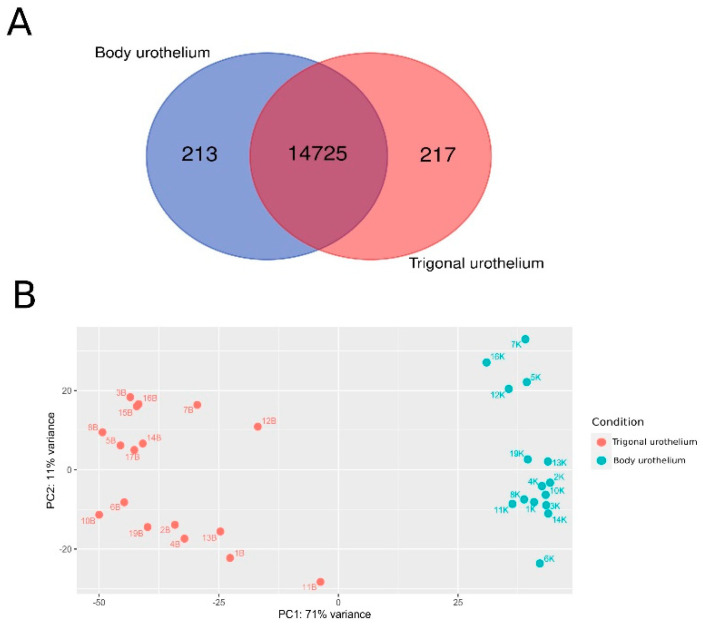
(**A**). Numbers of common and unique genes with noticeable levels of expression (greater than ten reads) in at least three urothelial samples. (**B**). Principal component analysis (PCA) based on transcriptomic profiling, showing clustering of the samples according to the origin of the urothelium, with B and K indicating trigonal and body urothelium samples, respectively.

To establish transcriptomic profiling, the total RNA samples isolated from the pairs of body and trigonal urothelia were sequenced, with the sequencing results for 17 trigonal samples and 15 body samples passing quality control and used for further analyses. The sequencing showed noticeable expression (i.e., with a base mean for genes of ten and greater than ten reads in at least three samples) of 14,938 genes from the body urothelia and 14,942 genes from the trigonal urothelia (Figure 2A). Principal component analysis (PCA) based on gene expression profiles (Figure 2B) showed that the sequenced samples clustered according to the source of urothelial samples. Pairwise comparisons between samples from the two urothelium locations identified 10,261 differentially expressed genes (DEGs) (*p*adj-value ≤ 0.05), with 5797 genes more highly expressed in body urothelium and 4464 genes more highly expressed in the trigonal urothelium.

### 3.2. Functional Annotations in the Reactome Database

Specific differences in gene expression profiles were identified using the Reactome database to annotate the functions of DEGs. Functional analyses showed that DEGs associated with 318 pathways were significantly upregulated (*p*adj-value ≤ 0.05) in the body urothelium, and that DEGs associated with 476 pathways were significantly upregulated (*p*adj-value ≤ 0.05) in the trigonal urothelium (Tables S1 and S2, respectively). Because these numbers precluded generalization of functional changes, the top 1000 DEGs upregulated in the body or trigonal urothelium were selected. Functional analyses showed that 36 pathways associated with the enrichment of at least ten DEGs were significantly upregulated in body epithelium and 77 pathways were significantly updregulated in the trigonal urothelium (Tables S3 and S4, respectively).

**Figure 3:**
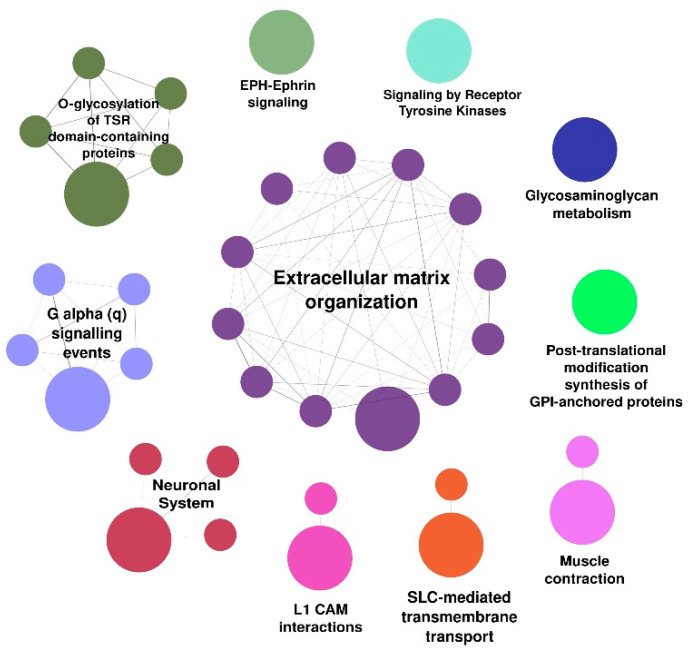
Summary of the Reactome categories and functional pathways enriched in bladder body urothelium (shown in Table S3). Big dots—category; small dots—pathways.

The Reactome-related pathways enriched in the body urothelium could be sorted into 11 categories, including *extracellular matrix organization* (consisting of 12 related pathways), *G alpha (q) signaling events* (five related pathways), *O-glycosylation of TSR domain-containing proteins* (five related pathways), *neuronal systems* (four related pathways), *muscle contraction*, *SLC-mediated transmembrane transport*, *L1CAM interactions*, and *glycosaminoglycan metabolism* (Figure 3). These pathways could be further categorized according to their protective functions by maintaining the integrity of the bladder epithelial barrier and regulatory functions of detrusor contractility integral to the neuronal system.

**Figure 4:**
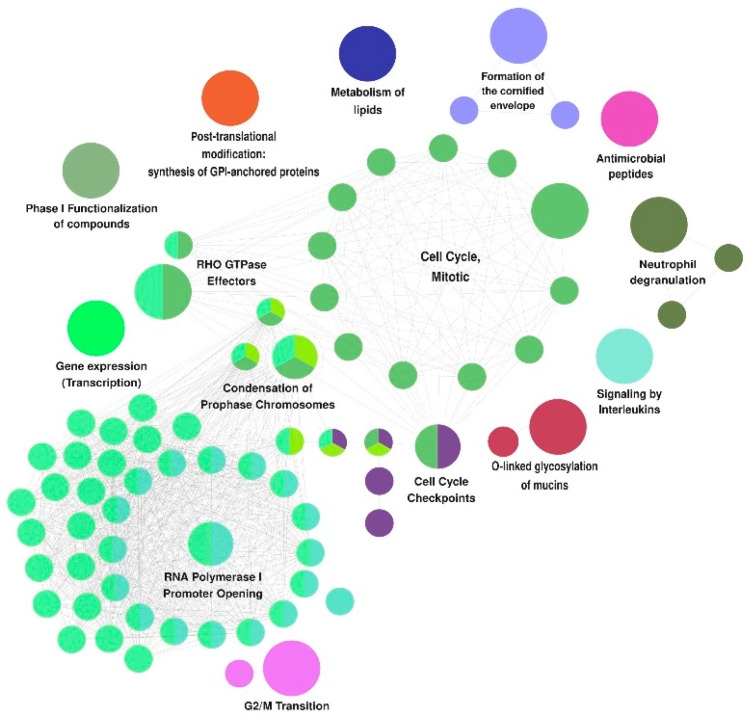
Summary of the Reactome categories and functional pathways enriched in trigonal urothelium (shown in Table S4). Big dots—category; small dots—pathways.

The Reactome-related pathways enriched in the trigonal urothelium could be assorted into 15 categories, including *RHO GTPase effectors* (consisting of 21 related pathways), *RNA polymerase I promoter opening* (18 related pathways), *cell cycle*, *mitotic* (13 related pathways), *cell cycle checkpoints* (five related pathways), *condensation of prophase chromosomes* (four related pathways), *formation of the cornified envelope* (three related pathways), *neutrophil degranulation* (three related pathways), and *G2/M transition* (two related pathways) (Figure 4).

In the trigonal urothelium, the category formation of the *cornified envelope* showed the highest statistically significant differences, with a *p*adj-value of 3.66 × 10^−26^, whereas the category *RHO GTPase effectors* was represented by the largest number of enriched pathways. Categories associated with the *cell cycle system*, including *cell cycle mitotic*, *cell cycle checkpoints*, and *G2/M transition*, were also significantly enriched in the trigonal epithelium. The categories *condensation of prophase chromosomes* and *RNA polymerase I promoter opening* showed significant differences in rates of proliferation between the body and trigonal epithelia. The pathways in the categories *neutrophil degranulation*, *immune system*, *signaling by interleukins*, and *antimicrobial peptides*, all of which were enriched in the trigonal epithelium, showed that urothelia at the two locations differed in their responses to immune stimuli.

Overall, these findings showed that body and trigonal urothelia differed significantly in their transcriptomic make-up responsible for maintaining bladder homeostasis. The transcriptome datasets were further explored by focusing on the selected genes that have been previously reported to be specifically expressed in human urothelium [7,18].

### 3.3. Differential Expression of Selected Genes

Uroplakin proteins form a highly specialized superstructure termed the asymmetric unit membrane (AUM) [34], and uroplakin 2 (UPK2) is the most highly expressed uroplakin protein in the umbrella cells of the bladder urothelium [35]. The present study found that the levels of expression of four uroplakin genes, *UPK1A*, *UPK1B*, *UPK2*, and *UPK3A*, were significantly higher (*p*adj-values ≤ 0.05) in the bladder body than in the trigonal urothelium, with FCs of 4.8, 15.1, 8.0, and 19.8, respectively.

**Figure 5:**
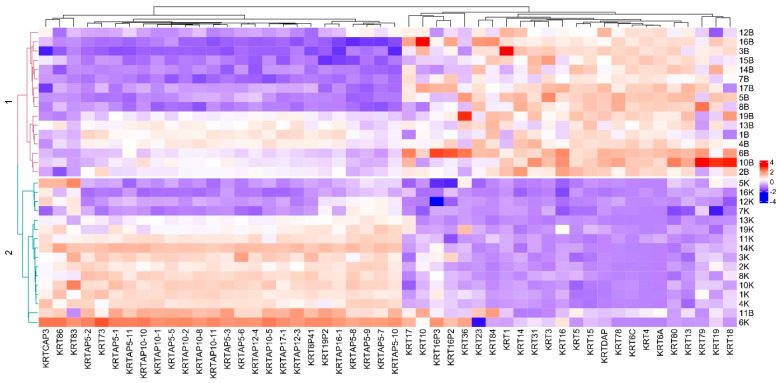
Heatmap of the expression of keratins in bladder body and urothelium samples. The expression of each gene was log-normalized for visualization using DESEQ2 function rld and scaled with an R function scale.

The analysis of keratin transcript levels revealed that, except for four bladder body urothelial samples, the unsupervised hierarchical clustering of the sequenced samples generally separated the bladder body and trigonal urothelium samples (Figure 5). PCA, however, separated these four bladder body urothelium samples from the other body urothelium samples (Figure 2B).

Acting as sensors, the urothelial receptors transduce signals coordinating interactions among the various cell types that form the bladder wall. Six of seven genes encoding members of the ATP-gated P2X receptor cation channel family were found to be more highly expressed in the body urothelium than in the trigonal urothelium (Table 2). In addition, five of six genes encoding members of the transient receptor potential channel family (*TRPA1*, *TRPM4*, and *7*, and *TRPV2* and *3*); the *CHRM4* gene encoding the muscarinic acetylcholine receptor M4; the *TH* gene encoding the rate-limiting enzyme in the synthesis of catecholamines; the VIPR1 gene encoding the vasoactive intestinal polypeptide receptor 1, were all more highly expressed in the bladder body than in the trigonal urothelium. Seven of eleven genes encoding C-C chemokines were also more highly expressed in the body urothelium, whereas 10 of 14 genes encoding C-X-C chemokine ligands or their receptors were more highly expressed in the trigonal urothelium. In addition, the expression of the *TAC1* (tachykinin precursor 1) gene, which encodes four products of the tachykinin peptide hormone family (substance P, neurokinin A, neuropeptide K, and neuropeptide gamma), was 47 times higher in the body than in trigonal urothelium (*p*adj-value = 5.49 × 10^−17^).

As reported previously, the pattern of nuclear estrogen receptor expression in the trigonal epithelium was similar to that reported in the vaginal epithelium [36,37]. The present study found that the expression of the estrogen receptor 1 (*ESR1*) gene was higher in the trigonal urothelium (*p*adj-value = 6.85 × 10^−95^; FC = 33.3 (fold change)), whereas the expression of the ESR2 gene was higher in the body urothelium (*p*adj-value = 4.78 × 10^−15^; FC = 25.1). The expression of the gene encoding progesterone receptor (*PGR*) was higher in trigonal urothelium (*p*adj-value = 2.31 × 10^−06^; FC = 8.3), whereas the expression of progesterone receptor membrane component 1 (*PGRMC1*) was higher in body epithelium (*p*adj-value = 8.0 × 10^−04^; FC = 1.52); the expression of *PGRMC2* was similar in both locations.

### 3.4. Mucosal Metagenomics

**Figure 6:**
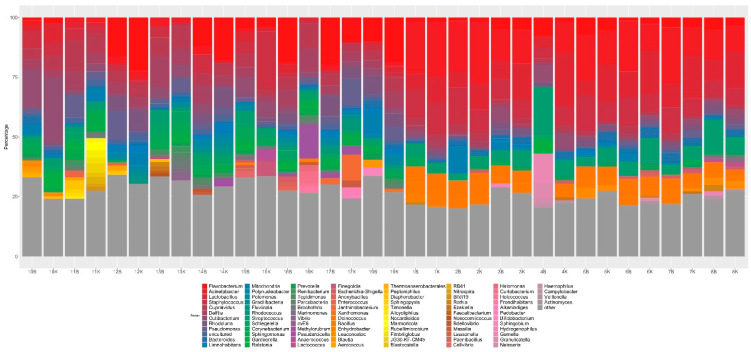
Genera of microbiomes isolated from bladder body (K) and trigonal (B) urothelium. Only the most abundant bacteria are shown.

Because bacterial deposits are present in the bladder epithelium [38], bacterial DNA was extracted from the bladder body and trigonal urothelial specimens, and bacterial 16S rRNA hyper-variable regions were sequenced using the PGM platform. The samples generated an average of 165,287 reads that passed the quality filtering thresholds. More than 99% of the reads in each sample could be classified into taxa. Of the 1451 taxa identified, averages of 4.78% and 1% could be classified as chloroplasts and mitochondria, respectively, and were therefore excluded from further analysis. The most abundant genera observed in both body and trigonal urothelial tissues were *Flavobacterium*, *Acinetobacter*, *Streptococcus*, *Lactobacillus*, and *Staphylococcus* (Figure 6). Although the abundance of 22 taxa, including genera with more than 10,000 reads such as *Microbacterium*, *Delftia*, *Staphylococcus*, *Bacteroides*, and *Saccharimonadales_ge*, differed in bladder body and trigonal urothelial samples (*p*-values ≤ 0.05); none of these differences was statistically significant after FDR *p*-value adjustment (Supplementary Table S5). There were also no statistically significant differences in indices of diversity, as shown by the non-parametric Shannon index (*p*-value = 0.2), or richness indices, as shown by the Chao1 index (*p*-value = 1).

### 3.5. Urine Metagenomics

Urinary bacterial profiles were compared in the 17 adolescent girls with pain and urological symptoms and their female family members (mothers and sisters). The urine samples generated an average of 148,213 reads that passed the quality filtering thresholds. More than 99.99% of the reads in each sample could be classified into 743 taxa, with *Lactobacillus*, *Streptococcus*, *Escherichia-Shigella*, *Peptoniphilus*, and *Gardnerella* representing the most abundant genera (Figure S1). After FDR correction, no statistically significant differences were observed between the female patients and their female family members (Supplementary Table S6). In addition, microbiome richness and diversity indices did not differ significantly in these sets of urine samples (Supplementary Table S7).

The urine samples collected from healthy adolescent female controls generated an average of 133,405 reads that passed the quality filtering thresholds. More than 99.99% of the reads in each sample could be classified into 634 taxa. Five of these taxa, including abundant taxa such as unclassified members of the *Comamonadaceae* *family* and *Aminobacter*, differentiated healthy controls from patients with urological symptoms. Presence/absence testing showed that two taxa, *Variovorax* and *Aminobacter*, were underrepresented, and seven were overrepresented in healthy controls (Table 3, Supplementary Table S8). Nevertheless, there were no statistically significant differences in diversity (non-parametric Shannon index *p*-value = 0.97) and richness (Chao1 index *p*-value = 0.13) indices between patients and controls.

## 4. Discussion

Morphological changes in the trigonal uroepithelium are often accompanied by a range of symptoms, including urinary frequency, urgency, urge incontinence, dysuria, and recurrent urinary tract infections. Trigonitis has been traditionally characterized as inflammation, ranging in intensity from minimal to severe, accompanied by non-keratinizing squamous metaplasia, although no unified histological definition has been determined [37]. In contrast, non-keratinizing squamous metaplasia, predominantly occurring in women in the trigone and bladder neck, is regarded as a normal variant of the urothelial mucosa [11,15,16].

To date, subtypes of bladder urothelia have been mostly studied by microscopic examinations, with molecular studies based on the expression of a small number of selected genes. To our knowledge, the present study is the first systematic comparison of genome-wide gene expression profiles in the bladder body and trigonal urothelia evaluated in adolescent female patients with micturition dysfunction.

Our transcriptomic data identified thousands of DEGs in urothelium samples from the bladder body and trigone, with hundreds of significant functional differences observed after annotation of these DEGs to Reactome database terms. The pathways enriched in the body urothelium included those representing the following: (1) *the organization of the extracellular matrix (ECM)* and *glycosylation of its glycosaminoglycans*; (2) *G-protein signaling*; and (3) *the neuronal system and muscle contraction*. The adhesive components of the ECM include collagens, fibronectin, and laminin, with the highly heterogeneous and dynamic composition of the ECM likely associated with the impermeability of the bladder body urothelium. The other categories enriched in the body urothelium are associated with the predominant functions of the bladder body, including sensory signaling leading to muscle detrusor contractility.

The pathways significantly enriched in the trigonal urothelium included those associated with *epithelial keratinocyte differentiation*, as represented by components of skin cornified envelope (the *formation of the cornified envelope* pathway). Other pathways enriched in the trigonal urothelium included those associated with changes in *cell proliferation* and *differentiation*, as represented by *RHO GTPases*, the *cell cycle*, and the *immune system*. The Rho family of GTPases is part of the Ras superfamily, which contributes to the organization of actin and micro-tubule cytoskeletons. These pathways are involved in the *regulation of vesicle trafficking*; *cell cycle progression*; *cell morphogenesis*, *polarity*, and *migration*; *inflammation*; and *wound repair* [39].

These findings were confirmed by analyses of the expression of selected genes that had been previously reported to be specifically altered in the human urothelium. These genes included those encoding uroplakins, keratins, and different types of urothelial receptors.

Uroplakins form urothelial plaques on the apical surface of the urothelium composed of terminally differentiated top umbrella cells, the cells primarily responsible for urothelial impermeability [10,11,19,20]. These umbrella cells, along with a mucopolysaccharide-rich layer of GAGs, form one of the strongest epithelial barriers in the human body [40,41]. The present study found that four of five uroplakins, *UPK1A*, *1B*, *2*, and *3A*, were highly expressed in the bladder body urothelium. Immunohistochemical assays have shown that uroplakin expression was reduced in intermediate cells and largely absent in basal cells [42,43,44].

The cytoskeletal structure consists of intermediate filaments (IF), which include various protein families, including keratins, a protein family encoded by 54 genes [45]. Keratins are highly expressed in the IF of epithelial cells [46] and represent nearly 5% of the total cell proteome. These proteins direct structural organization in response to mechanical stress and are therefore responsible for the integrity and mechanical stability of the epithelium [47]. The present study showed that the levels of transcripts encoding keratins could differentiate samples from the bladder body and trigonal urothelia, with four of these, K5, K13, K18, and K19, differing significantly between the two bladder locations with FCs higher than five. Previous microscopic-based analyses found that in non-stratified (simple) epithelia, which experience less mechanical stress, only a few keratins, including K8/K18 and K19, form sparse and loosely distributed keratin filaments in the cytoplasm. In contrast, many additional keratins, including K1, K2, K5, K10, and K14, were found to be present in the cytoskeleton of cornified squamous epithelia [46,48].

The *formation of the cornified envelope* pathway was found to be one of the most significantly enriched pathways in the trigonal urothelium, indicating the possible consequences of contact between bladder urothelium and urine. Although the corpus urothelium is primarily exposed to urine hydrostatic pressure, the bladder base, consisting of the trigone and bladder neck, may also experience damage from urine flow. Therefore, a *cornified envelope* may serve as the first line of defense against environmental insults, not only in the skin, but also in other epithelia at risk of damage, including the oral cavity, esophagus, vagina, and bladder. The highly upregulated pathways in the trigonal urothelium were also annotated to categories associated with *cytoskeletal organization*; *cell morphogenesis*, *proliferation*, and *differentiation;* and *inflammatory processes.*

The functions of the bladder, urethra, and urethral sphincter are strictly coordinated by urothelial receptors and channels, which act as sensors and transduce signals from the urothelium to the suburothelial nerve plexus, the interstitial cells of Cajal, detrusor smooth muscle cells, and immune cells [49,50,51]. Purinergic, adrenergic, cholinergic, protease-activated, neuropeptide, and chemokine receptors, along with ion and transient receptor potential channels, have been found to respond to various stimuli, such as bladder stretching and distension, soluble factors, chemokines, and changes in urinary pH [7]. The present study showed that the expression of most urothelial sensors was higher in the body than in trigonal urothelium, including genes encoding *P2X receptors*, *TRP channels*, *C-C chemokines*, *CHRM4*, *TH*, and *VIPR1*. P2X receptors are membrane ion channels, activated by the binding of extracellular adenosine triphosphate (ATP), that transduce afferent and chronic pain signaling in the bladder [52,53]; TRP channels are nonspecific cation channels that are permeable to Ca^2+^ and might act as sensors of stretching and/or chemical irritation [54]; the *CHRM4* gene encodes the muscarinic acetylcholine receptor M4; the *TH* gene encodes the rate-limiting enzyme in the synthesis of catecholamines; and the *VIPR1* gene encodes vasoactive intestinal polypeptide receptor 1. In contrast, the expression of genes encoding C-X-C chemokines and their receptors was higher in trigonal than in bladder body urothelium. Members of the C-X-C family of chemokines (CXCL1 to 17) and six of their receptors attract neutrophils and lymphocytes and promote immune responses and stem-cell survival, development, and homeostasis, triggering chemotaxis and angiogenesis. They play roles in normal and pathological processes, including allergic responses, infectious and autoimmune diseases, inflammation, and angiogenesis [55,56]. Most of the main pathologic conditions of the bladder, including cancer, urinary tract infections, and BPS/IC, may significantly affect the expression of urothelial receptors and channels, resulting in micturition dysfunctions [42,50].

Quantitative real-time-PCR-based gene expression profiling has shown that the levels of mRNAs encoding *CHRM* and *3*, *P2X1*, *NK2R*, and *ASIC1a* were significantly higher in the bladder body than in the trigone, but that the levels of the uroplakin *UPK2* mRNA did not differ significantly [17].

The trigonal urothelium may preferentially respond to estrogen stimulation, as its estrogen receptor expression pattern is similar to that reported in the vaginal epithelium [36,37]. The present study confirmed that mRNAs encoding estrogen and progesterone receptors were differentially expressed in the bladder body and trigonal urothelium.

Although this study provides comprehensive information on molecular differences associated with the distinct functions of the bladder body and trigonal urothelia, the cause-and-effect relationships between clinical symptoms and molecular changes have not been determined. Similar clinical symptoms may occur in patients with different pathologies, making patient selection particularly difficult. The pediatric patients included in this study appeared to be histopathologically heterogeneous, with the microscopic changes in urothelium ranging from borderline normal to chronic inflammation and clinical symptoms ranging from weak to moderate in intensity. In addition, some patients had non-keratinizing squamous metaplasia, whereas others did not, with some specimens having non-keratinizing squamous metaplasia alone, without any other changes.

To determine if the clinical symptoms in this patient cohort were related to chronic urinary tract infections, the bacterial profiles in urine and epithelium were analyzed by sequencing bacterial 16S rRNA hyper-variable regions. The cystoscopy in postmenopausal women with recurrent urinary tract infections and fulguration of trigonitis showed that bacteria formed communities within the bladder epithelium, accompanied by active adaptive cellular and humoral immune responses [9]. In addition, squamous metaplasia may predispose one to chronic infection [15]. However, although all patients experienced episodes of urinary infections during early childhood, none had urinary tract infections during the study visit, as determined by urinary cultures and sequencing-based analyses. In addition, bacterial microbiomes of the body and trigonal urothelia did not differ significantly. Thus, although infection could have initiated micturition disorders in these patients, infections did not accompany their bladder symptoms. In contrast, transcriptomic analyses showed overexpression of genes involved in the antimicrobial peptide (AMP) pathway, including *BPIFB1*, *CAMP*, *CHGA*, *DEFB1*, *LCN2*, *PGLYRP2*, *PGLYRP3*, *PGLYRP4*, *PI3*, *PRSS3*, *RNASE7*, *S100A7*, and *S100A9*. Because epithelial and immune cells are constantly exposed to microbes, secreted AMPs, including defensins, cathelicidins, and lipocalins, are essential for barrier defense, with deficiencies in AMPs leading to infection [57].

A suprapubic pain related to bladder filling in patients with BPS/IC was found to be unaccompanied by a proven urinary infection or other obvious pathology of the lower urinary tract [58]. In contrast, the expression of many proteins involved in cellular differentiation, barrier function, and bacterial defense mechanisms was shown to be altered in the urothelia of these patients [7,59,60,61,62]. Similarly, single-cell transcriptomes of mouse bladder urothelium identified several BPS/IC-regulating genes differentially expressed among different urothelial cell subpopulations [49], with incomplete urothelial differentiation characterized by reduced expression of uroplakins and the absence of urothelial plaques in the apical plasma membrane, leading to a “leaky” barrier [12,13,14]. Together with changes in the expression of sensory proteins, abnormal uroplakin expression is a unifying hallmark of BPS/IC [42]. However, the lack of significant differences in keratinocyte differentiation, as shown by primary cultures of urothelial biopsies taken from both IC and normal urothelium, is in disagreement with the theory of global and intrinsically abnormal keratinocyte differentiation in BPS/IC [63].

## 5. Conclusions

Most of our female patients with micturition dysfunction presented clinical symptoms characterized by the complaint of suprapubic pain related to bladder filling with co-existing lower urinary tract symptoms, such as urgency and increased daytime and nighttime frequency, while the urodynamic examination diagnosed the detrusor overactivity with increased sensation in the bladder. Both urine culture and metagenomics analyses found no evidence for persistent urinary infection, whereas transcriptional data revealed the activation of inflammatory pathways in the absence of histopathologic evidence of predominant inflammation of the trigonal urothelium. Collectively, these findings suggest that our patients can be predominantly regarded as having a type of bladder disorder called BPS/IC.

Understanding the molecular mechanisms of disease requires molecular diagnostics, and collecting, cataloging, and comparing data from molecular studies create the fundamentals for biomarker discovery. However, despite molecular studies that have been conducted in BPS/IC, neither disease biomarkers which could aid in diagnosis nor potential targets for treatment have been identified yet [64]. Many aspects of BPS/IC, including pathophysiology, proper causative definitions as well as diagnostic and treatment criteria remain to be clarified [65,66]. Unfortunately, our study did not determine an association of molecular findings with clinical symptoms. For such analyses, we would have needed to collect transcriptional data from the urothelia of healthy adolescents which, following the ethical limitations, was prevented. In addition, although bladder dysfunction in Polish patients occurs almost exclusively in girls [24], further studies should also include male pediatric patients, and molecular studies should be repeated in more diverse groups of patients.

## Figures and Tables

**Table 2:** Relative levels of expression of individual genes selected from the transcriptome data previously reported as being specifically expressed in human urothelium [7,18].

Upregulated in Trigonal Urothelium			Upregulated in Body Urothelium		
Gene	*p*adj-Value	FC	Gene	*p*adj-Value	FC
CXCL1	1.57 × 10^−49^	386.85	TAC1	5.49 × 10^−17^	47.38
CXCL6	1.67 × 10^−63^	70.71	CCL15	1.15 × 10^−33^	16.37
CXCL3	1.00 × 10^−15^	34.22	TH	3.75 × 10^−08^	6.80
CXCL2	1.02 × 10^−15^	28.83	P2RX2	3.24 × 10^−02^	5.48
CXCR1	4.20 × 10^−05^	9.46	TRPV3	1.86 × 10^−12^	5.43
CALCA	1.93 × 10^−02^	8.08	TRPA1	1.82 × 10^−11^	5.22
CXCL17	1.69 × 10^−25^	7.51	VIPR1	1.40 × 10^−15^	4.99
CCL22	1.92 × 10^−11^	6.33	CCL13	3.21 × 10^−04^	4.39
CCL17	2.95 × 10^−14^	6.05	CCL25	1.05 × 10^−02^	4.30
CXCR2	4.18 × 10^−27^	5.77	CCL16	4.54 × 10^−02^	3.96
CCL7	1.12 × 10^−02^	5.12	CCL27	8.98 × 10^−04^	3.71
P2RY2	2.57 × 10^−17^	3.76	CCL14	1.10 × 10^−03^	3.69
TRPM6	7.46 × 10^−04^	2.95	CHRM4	8.34 × 10^−05^	3.58
CCL2	8.29 × 10^−06^	2.89	P2RX6	3.51 × 10^−05^	3.40
P2RY1	4.45 × 10^−06^	2.52	CXCL12	4.10 × 10^−07^	3.20
CXCL10	1.07 × 10^−02^	1.95	CCL18	4.73 × 10^−02^	2.63
CXCL16	4.22 × 10^−04^	1.74	P2RX4	4.08 × 10^−15^	2.59
CX3CL1	7.44 × 10^−03^	1.61	CXCR2P1	1.26 × 10^−02^	2.41
			CXCR7	2.31 × 10^−05^	2.36
			P2RY4	1.01 × 10^−02^	2.27
			P2RX1	1.80 × 10^−02^	2.18
			P2RX5	3.50 × 10^−02^	2.15
			P2RY8	1.84 × 10^−02^	2.03
			TRPV2	8.50 × 10^−04^	1.81
			CXCR4	3.51 × 10-^02^	1.50
			TRPM7	5.16 × 10^−05^	1.40
			P2RX7	3.80 × 10^−02^	1.40
			TRPM4	4.04 × 10^−02^	1.38

FC; Fold Change.

**Table 3:** Presence/absence and abundance differences of bacteria, as determined by Fisher’s exact test and metagenomeSeq.

No. of Control Samples	No. of Patient Samples	FisherP	FisherAdjP	logFC	se	*p*-Value	*p*adj-Value	Size	Taxonomic Classification
0	11	1.83 × 10^−08^	1.66 × 10^−05^	3.81	0.40	0.00	0.00	60,407	*Aminobacter (100)*
12	10	1.99 × 10^−01^	1.00 × 10^00^	3.92	0.66	2.12 × 10^−09^	9.59 × 10^−07^	51,881	*Comamonadaceae_unclassified (100)*
0	7	1.62 × 10^−04^	2.45 × 10^−02^	1.29	0.29	5.94 × 10^−06^	1.79 × 10^−03^	2783	*Variovorax (100)*
13	4	1.32 × 10^−01^	1.00 × 10^00^	−4.52	1.05	1.85 × 10^−05^	4.19 × 10^−03^	855	*Eukaryota_unclassified(100)*
16	1	9.04 × 10^−05^	2.45 × 10^−02^	−1.53	0.39	9.09 × 10^−05^	1.65 × 10^−02^	1440	*Cupriavidus (100)*
15	0	5.00 × 10^−05^	2.27 × 10^−02^	−1.82	0.53	5.58 × 10^−04^	7.22 × 10^−02^	22,283	*Mitochondria_ge (100)*
13	0	3.17 × 10^−04^	3.60 × 10^−02^	−1.53	0.44	5.27 × 10^−04^	7.22 × 10^−02^	1344	*Mesorhizobium (100)*
13	0	3.17 × 10^−04^	3.60 × 10^−02^	−1.44	0.42	6.66 × 10^−04^	7.53 × 10^−02^	1520	*Amaricoccus (100)*
19	4	1.62 × 10^−04^	2.45 × 10^−02^	−0.45				443,343	*Gardnerella (100)*
19	4	1.62 × 10^−04^	2.45 × 10^−02^	−2.99				8891	*Muribaculaceae_ge (100)*

FisherP, FisherAdjP—*p*-value and adjusted *p*-value in Fisher’s exact test for presence/absence of taxa in samples; logFC—logarithm with base 2 from fold-change; *p*-value, *p*adj-value—*p*-value for differential bacterial abundance testing in metagenomeSeq model; Size—total number of reads.

## Data Availability

The RNA-Seq and 16S rRNA sequencing data have been deposited as BAM files in the European Nucleotide Archive (Accession number: PRJEB50164).

## Supplementary Materials

The following supporting information can be downloaded at: https://www.mdpi.com/article/10.3390/biomedicines10061435/s1. Supplementary Table S1. Reactome related pathways enriched in the body urothelium. Supplementary Table S2. Reactome related pathways enriched in the trigonal urothelium. Supplementary Table S3. Reactome related pathways enriched in the body urothelium. Bolded—overriding categories. Supplementary Table S4. Reactome related pathways enriched in the trigonal urothelium. Bolded—overriding categories. Supplementary Table S5. Results of mixed effects models of comparison between healthy and metaplastic tissue. OTU—OTU number, Estimate—beta coefficient in mixed effects model, t.value—test statistic for a coefficient, *p*-value—*p*-value for coefficient’s significance, Size—number of reads assigned to an OTU, Taxonomy—OTU taxonomy, *p*adj—*p*-value after FRD correction. Supplementary Table S6. Results of mixed effects models of comparison between patients and their mothers. OTU—OTU number, Estimate—beta coefficient in mixed effects model, t.value—test statistic for a coefficient, *p*-value—*p*-value for coefficient’s significance, Size—number of reads assigned to an OTU, Taxonomy—OTU taxonomy, *p*adj—*p*-value after FRD correction. Supplementary Table S7. Mann Whitney U-test results between family members’ urine richness and diversity indices. Supplementary Table S8. Fisher’s exact test results for differential presence/absence of taxa and zero-inflated log-normal model for differential abundance in comparison between healthy controls and metaplastic patients. Supplementary Figures: Supplementary Figure S1. Genera of microbiomes isolated from urine samples obtained from patients with urological symptoms and female family members (Panel A) and from patients and female controls (Panel B). Unclassified reads on this level were not included. Only the most abundant bacteria are shown.
