# Supplementary figures and images for: Gene Expression-Based Functional Differences between the Bladder Body and Trigonal Urothelium in Adolescent Female Patients with Micturition Dysfunction
Source: Biomedicines. 2022 Jun 17;10(6):1435. doi: 10.3390/biomedicines10061435 (PMC9220714; doi:10.3390/biomedicines10061435)

A

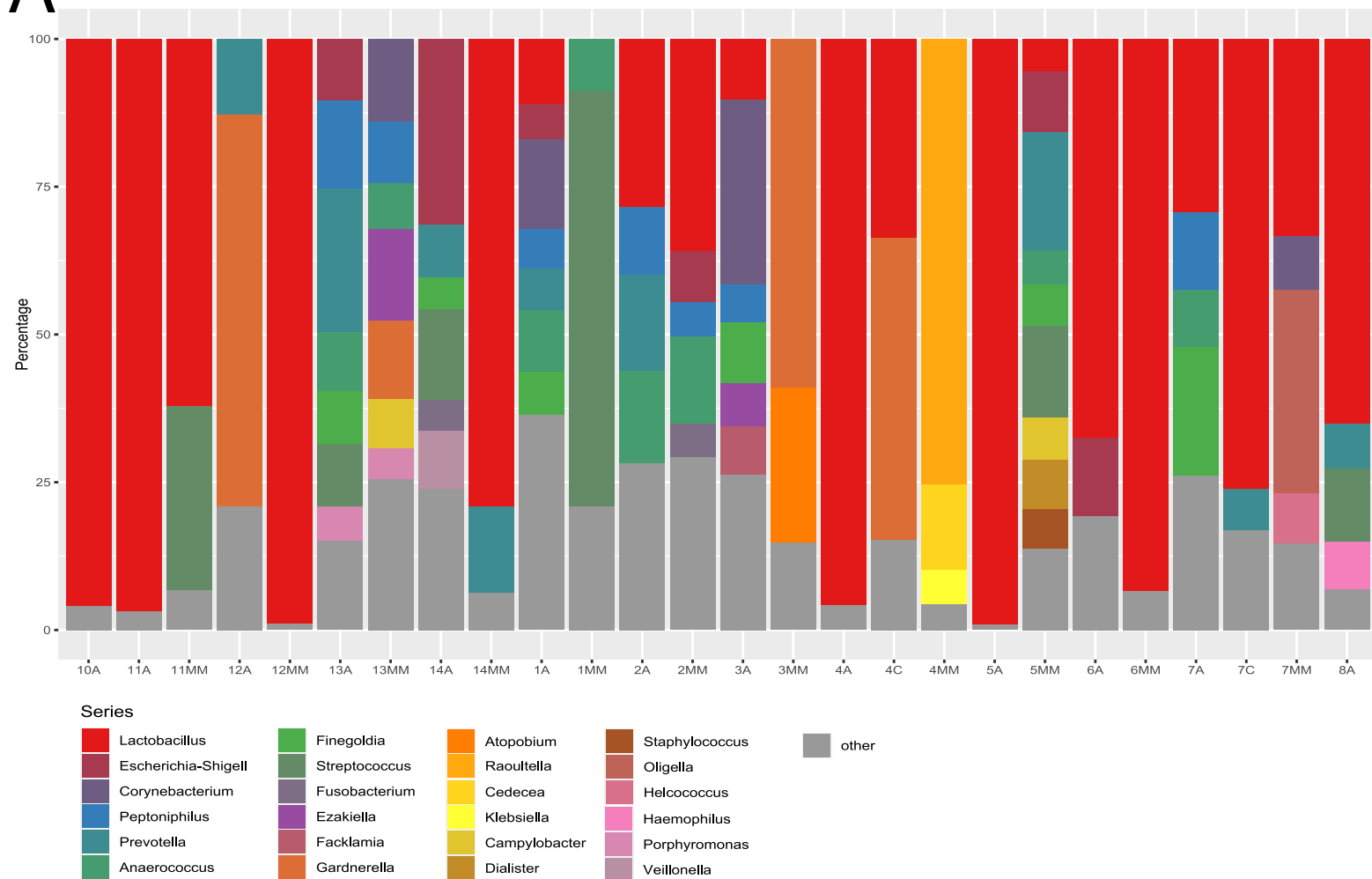

B

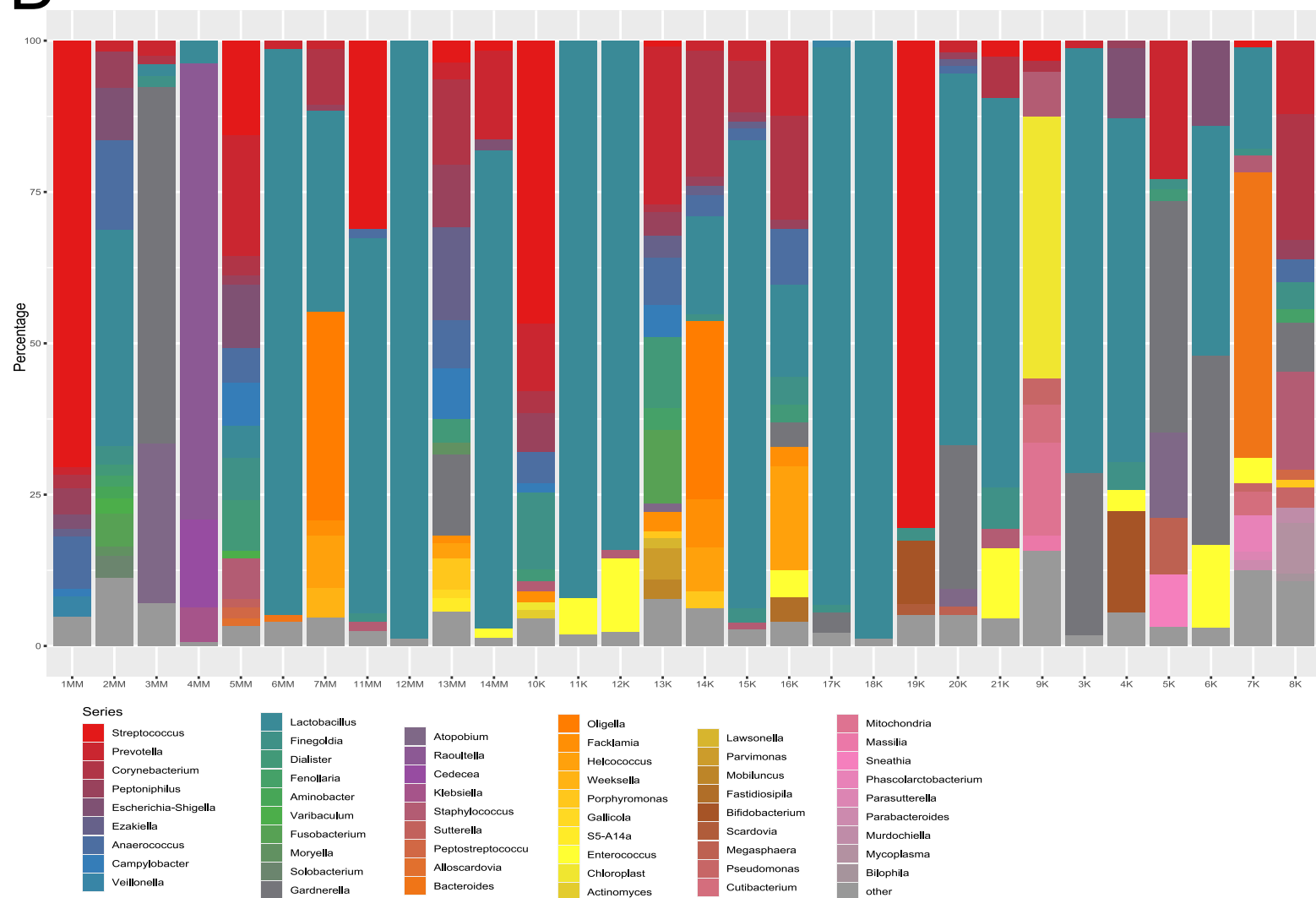

Supplement: Supplementary file 1 [file biomedicines-10-01435-s001.zip › biomedicines-1740602-Supplementary Material/biomedicines-1740602-suppl figures.pdf]
